# Genome-wide analysis of epigenetic and transcriptional changes in the pathogenesis of RGSV in rice

**DOI:** 10.3389/fpls.2022.1090794

**Published:** 2023-01-11

**Authors:** Xiaoqing Wu, Hongfei Liu, Bi Lian, Xue Jiang, Cheng Chen, Tianxin Tang, Xinlun Ding, Jie Hu, Shanshan Zhao, Shuai Zhang, Jianguo Wu

**Affiliations:** ^1^ Vector-borne Virus Research Center, Key Laboratory of Plant Virology of Fujian Province, Institute of Plant Virology, College of Plant Protection, Fujian Agriculture and Forestry University, Fuzhou, China; ^2^ Center for Plant Biology, School of Life Sciences, Tsinghua University, Beijing, China

**Keywords:** association analysis, RGSV, methylation, transcriptome, H3K9me3

## Abstract

Rice grassy stunt virus (RGSV), a typical negative single-stranded RNA virus, invades rice and generates several disease signs, including dwarfing, tillering, and sterility. Previous research has revealed that RGSV-encoded proteins can force the host’s ubiquitin-proteasome system to utilize them for viral pathogenesis. However, most of the studies were limited to a single omics level and lacked multidimensional data collection and correlation analysis on the mechanisms of RGSV-rice interactions. Here, we performed a comprehensive association analysis of genome-wide methylation sequencing, transcriptome sequencing, and histone H3K9me3 modification in RGSV-infested as well as non-infested rice leaves, and the levels of all three cytosine contexts (CG, CHG and CHH) were found to be slightly lower in RGSV-infected rice leaves than in normal rice. Large proportions of DMRs were distributed in the promoter and intergenic regions, and most DMRs were enriched in the CHH context, where the number of CHH hypo-DMRs was almost twice as high as that of hyper-DMRs. Among the genes with down-regulated expression and hypermethylation, we analyzed and identified 11 transcripts involved in fertility, plant height and tillering, and among the transcribed up-regulated and hypermethylated genes, we excavated 7 transcripts related to fertility, plant height and tillering. By analyzing the changes of histone H3K9me3 modification before and after virus infestation, we found that the distribution of H3K9me3 modification in the whole rice genome was prevalent, mainly concentrated in the gene promoter and gene body regions, which was distinctly different from the characteristics of animals. Combined with transcriptomic data, H3K9me3 mark was found to favor targeting highly expressed genes. After RGSV infection, H3K9me3 modifications in several regions of CTK and BR hormone signaling-related genes were altered, providing important targets for subsequent studies.

## Introduction

Plants have evolved several different molecular mechanisms to resist viruses, including R genes and recessive resistance, RNA silencing, phytohormone signaling, autophagy, and WUS-mediated antiviral immunity. Recent studies have found that the methylation levels of host plant-encoded genes are reshaped in response to viral infection, linking plant antiviral silencing to epigenetic modifications of genomic DNA and proteins. Epigenetic regulatory mechanisms, including DNA methylation, histone modifications and some non-coding RNA (NcRNA) change, affect chromatin structure even in the absence of cross-genomic mutations, which provide the molecular memory that leads to abnormal development in plants and mammals (Quadrana and Colot, 2016; Lang et al., 2017; Dong et al., 2018).

DNA methylation, defined as the covalent attachment of a methyl group to the cytosine 5 carbon position of a genomic CpG dinucleotide by DNA methylation transferase, is a chemical modification that can affect genetic expression without changing the DNA sequence and is an important mechanism of controlling gene expression at the epigenetic level. According to the sequence context of methylation modifications, DNA methylation is mainly divided into CG, CHG and CHH types (H stands for A \ T \ C), and these three methylation modifications depend on the action of Domains Rearranged Methyltransferase 2 (DRM2) (Cao and Jacobsen, 2002; Law and Jacobsen, 2010). In Arabidopsis, Methyltransferase 1 (MET1) maintains CG type methylation (Ronemus et al., 1996), Chromomethylase 3 (CMT3) maintains CHG site methylation (Cao and Jacobsen, 2002), DRM2 and CMT2 maintains CHH type methylation (Shen et al., 2014). Among them, CHH-type *de novo* methylation relies on the RNA-dependent DNA methylation (RdDM) pathway. First, the RNA Polymerase IV (Pol IV) protein transcribes DNA into a single-stranded RNA sequence, RDR2 converts the single-stranded RNA into a double-stranded RNA sequence, and then DCL3 cuts it into a 24-nt siRNA sequence. The siRNAs of these 24-nt bind to AGO4 proteins to form silent complexes that specifically target RNA Polymerase V (Pol V) transcripts and recruit DRM2 to unmethylated homologous genomic sites for methylation (Chan et al., 2004; Jia et al., 2009; Havecker et al., 2010; Matzke and Mosher, 2014). CHG and CG methylation mainly regulate heterochromatin formation and transcriptional gene silencing, while CG methylation mainly occurs within genes to regulate gene expression (Sijen et al., 2001; He et al., 2011; Melnyk et al., 2011).

DNA methylation is a crucial plant defense mechanism against DNA viral infection, particularly in the case of single-stranded DNA viruses (specifically geminiviruses), and this topic has been widely covered in the scientific literature. The host plant inhibits viral replication by elevating the methylation level of the virus genome, and so resists virus infection. At the same time, the virus employs its own encoded protein to disrupt the host plant’s defensive response (Raja et al., 2008; Yang et al., 2011; Zhang et al., 2011; Butterbach et al., 2014; Castillo-González et al., 2015; Gui et al., 2022). The Arabidopsis DNA methylation deletion mutant impaired its resistance to geminivirus and had more severe symptoms after infection, and the methylation level of viral DNA dropped. The viral DNA and associated histones are methylated in the diseased plant, demonstrating that methylation of the virus genome is an essential method for plants to resist Geminivirus infection at the epigenetic level (Raja et al., 2008). Similar to this, by increasing the methylation level of the viral genome, the disease resistance gene Ty-1 may mediate resistance to the Tomato Yellow Leaf Curl Virus (TYLCV) (Butterbach et al., 2014). However, in order to combat the defense mechanism of the host plant, the virus has evolved complex regulatory processes to reduce the level of methylation of the virus genome. The C2 protein of Beet Severe Curly Top Virus (BSCTV) interacts with S-Adenosyl-Methionine Decarboxylase (SAMDC) to weaken SAMDC1 degradation in cells, unbalance SAM/dcSAM levels, and eventually impede DNA methylation-mediated gene silencing of viral DNA and enhance virus replication (Zhang et al., 2011). βC1 encoded by satellite DNA of Tomato Yellow Leaf Curl China Virus (TYLCCNV) can interact with S-Adenosine Homocysteine Hydrolase (SAHH), a key enzyme in methylation modification, to inhibit its protease activity, resulting in a lower methylation level of the entire TYLCCNV genome and encouraging the expression of the virus genome (Yang et al., 2011). According to recent research, βC1 may also boost the glycosylase activity of Demeter (DME) by interaction, which diminishes the methylation level of viral DNA and stimulates virus infestation (Gui et al., 2022). The TrAP protein encoded by Tomato Golden Mosaic Virus (TGMV) and Cabbage Leaf Curl Virus (CaLCuV) interacts with H3K9me2 histone methyltransferase Keyptonite (KYP) protein to inhibit its methyltransferase activity and weaken the level of post-transcriptional gene silencing on virus chromosomes (Castillo-González et al., 2015). AGO4, a crucial component of the RdDM pathway, and the viral protein V2 encoded by Cotton Leaf Curl Multan Virus (CLCuMuV) and Tomato Yellow Leaf Curl Virus (TYLCV) interact to block AGO4 from binding to viral genomic DNA, dampening the viral genome’s methylation level (Wang et al., 2019b).

H3K9me3 is a repressive histone modification that has a very important role in mammalian embryonic development and cell fate determination as one of the markers of heterochromatin (Xu et al., 2022). H3K9me3 modifications are abundantly present in retrotransposon and some gene promoter regions in animal cells, which is generally regarded as barriers to intercellular fate transition (Wang et al., 2018). Most studies have linked H3K9me3 to gene transcriptional repression, and a few works have also found that H3K9me3 can also lead to gene transcriptional activation. Vakoc et al. (2005) found that H3K9 dimethylation and trimethylation occur in the transcribed regions of active genes in mammalian chromatin, and this modification is dynamic because it increases during transcriptional activation and is rapidly removed upon gene repression. Heterochromatin Protein 1γ (HP1γ), a protein containing a chromo-domain that recognizes H3K9 methylation, is also present in the transcribed region of all active genes examined. The binding of H3K9me3 and HP1γ was shown to be associated with gene transcriptional activation.

To resist host plant resistance, viral proteins also interfere with the expression of methylation pathway genes at the transcriptional and protein levels, thereby inhibiting host plant DNA methylation, changing the expression of methylation modification-related genes, and finally boosting virus infection and starting to cause an obvious pathogenic phenotype. Geminiviruses wreak havoc on host DNA methylation by lowering the transcriptional levels of plant DNA methyltransferases MET1 and CMT3, which are crucial for maintaining symmetric methylation. Rep (Replication associated protein) is the principal viral protein that inhibits DNA methyltransferase maintenance and reverses transcriptional gene silencing (TGS) of the host gene. It is believed that plant viruses have evolved a variety of independent strategies to overcome this host defense response (Rodríguez-Negrete et al., 2013). The viral repressor HCPro of Tobacco vein banding mosaic virus (TVBMV) stimulates the expression of YC1, YC5, and YC10 genes by lowering DNA methylation levels at their promoter regions, resulting in the buildup of auxin (Yang et al., 2020). Combined with transcriptome and genome-wide methylation sequencing data, it was discovered that in rice infected with the Rice Black-Streaked Dwarf Virus (RBSDV), the methylation and transcription level of many genes are correlated, and the expression of some genes involved in methylation modification is modified, such as the expression of DNA Methyltransferase 702 (OsDMT702) is suppressed and the expression of OsDMT704, OsDMT707, RNA-dependent RNA Polymerase 1 (OsRDR1) and OsRDR6 is enhanced. It is anticipated that the methylation alteration of the rice genome may have a substantial function in the virus-rice interaction (Li et al., 2018). Rice grass stunt virus (RGSV) is a Bunyavirales negative-strand RNA virus. RGSV pathogenic protein P3 can induce a U-box type E3 ubiquitin ligase P3-INDUCIBLE PROTEIN 1 (P3IP1) to target for degradation of Nuclear RNA Polymerase D1a (OsNRPD1a or Pol IVa), a key factor in the RdDM pathway, resulting in the formation of disease symptoms, including dwarfing and tillering in rice plants. It has been proposed that RGSV regulates the methylation level of the rice genome (Zhang et al., 2020).

Herein, we will apply epigenomics and transcriptomics to more precisely and systematically dissect the mechanism of action of RGSV causing dwarfism, multiple tillering and low fertility in rice. Through genome-wide methylation and transcriptome association analysis, we excavated and identified 18 transcripts related to fertility, plant height and tillering. By analyzing changes in histone H3K9me3 modification after viral infestation, we found that CTK and BR hormone signaling-related genes were closely related to H3K9me3 modification, which provided important targets for subsequent studies.

## Materials and methods

### Plant materials and growth conditions

Wild-type rice (NPB) plants grow in a greenhouse with 26°C, 70% relative humidity and a photoperiod of 14/10 (day and night). In order to obtain RGSV-infected plants, the pathogenic larvae of the brown planthopper (*Nilaparvata lugens*) carrying RGSV were inoculated with 14-day-old rice seedlings for 3 days, and then the inoculated rice plants were planted in greenhouses.

### DNA extraction and MethylC-seq library generation

Total genomic DNA was isolated from 0.5 g of rice leaves using the cetyl trimethyl ammonium bromide (CTAB) methods (Doyle, 1990), crushed in liquid nitrogen, treated with RNase I (10 U/ml) (Thermo Fisher Scientific), and purified. Purified genomic DNA (2.0 µg) was then utilized to create MethylC-seq libraries according to the method reported by Li et al. (Li et al., 2017). The resultant libraries were pooled and sequenced (single-end 50 bp, SE50) on a HiSeq 2500 machine (Illumina).

### BS-seq data processing and analysis

Use FastQC software (version 0.11.2; http://www.bioinformatics.babraham.ac.uk/projects/fastqc/) to check the quality of the original data, and then use Trimmomatic software (version 0.32; http://www.usadellab.org/cms/?page=trimmomatic) to carry out quality control (QC) of the original data. After removing the contamination of the PCR junction and the unmatched reads containing unknown bases (N), and retaining the high-quality sequence, the clean reads were obtained. Use BISMARK’s (version 0.12.5) default parameter to compare clean data to the reference genome of rice (MSU7, http://rice.uga.edu/), and remove repeated clips produced by PCR from each methylation group. The degree of methylation of each cytosine on the genome was then calculated [methylation percentage = C/(C+T)]. Considering sequencing depth and error, we require that cytosine be covered by at least 4 reads to be considered.

### DNA methylation difference region analysis

Use the software swDMR, set the parameters to divide the rice genome into windows with 200 bp length, and divide the 100 bp long step into bin, and calculate the average level of each methylation type (CG, CHG, CHH). Requires CG greater than or equal to 0.4, CHG greater than equal to 0.3, CHH greater than equal to 0.1, and FDR less than or equal to 0.01 deemed to be a differentiated bin. At the same time, it is required that each cytosine be covered by at least four sequencing reads, that there are at least four homologous methylation sites in each bin, that the DMR length cannot be 50 bp and that a distance is less than or equal to 100 bp is considered to be in the same DMR. The starting point and terminal of the resulting DMR are the first and last effective positions of cytosine (Xu et al., 2020).

### Total RNA extraction and QRT-PCR

The RNeasy Plant Mini kit (Qiagen) was used to extract total RNA in accordance with the manufacturer’s instructions. Using TransScript First-Strand cDNA Synthesis SuperMix (TransGen Biotech), the first strand of cDNA was generated and then utilized as a template for RT-PCR or qRT-PCR. Normalization of the relative expression level of a target gene to that of ACTIN. All qRT-PCR primers are published online in **Table S1**.

### mRNA-seq data processing

For RNA sequencing, the RNA quantity and purity were determined by the Novogene Bioinformatics Institute (Beijing, China). Use FastQC software (version 0.11.2; http://www.bioinformatics.babraham.ac.uk/projects/fastqc/) to check the quality of the original data, and then use the Trimmomatic software (version 0.32; http://www.usadellab.org/cms/?page=trimmomatic) to carry out quality control (QC) of the original data, and get clean reads after removing the low-quality sequence. Use HISAT2 software (https://ccb.jhu.edu/software/hisat2/index.shtml) default parameter settings to map clean data to the rice reference genome (MSU7, http://rice.uga.edu/); use the SAMTools software (version 0.1.18) to convert the SAM file to the BAM file, and use the built-in program to sort the results; Then use StringTie software (version 1.3.4d) to assemble the sorted transcripts, and finally use StringTie’s built-in Python program prepDE.py to calculate read counts through coverage values in GTF to get the expression matrix of genes and transcripts. The differences of the data are analyzed by R language (version 3.1.0), edgeR package (version 3.6.4), GO annotation and enrichment analysis are carried out by online website agriGOv2 (version 2.0; http://systemsbiology.cau.edu.cn/agriGOv2/index.php), GO enrichment map and heat map are drawn by R language, and Wayne diagram is drawn by TBtools.

### Protein extraction and western blot assay

Individually selected plant tissues were crushed in liquid nitrogen and subsequently homogenized in a protein extraction buffer (Sigma-Aldrich, Missouri, USA) containing a protease inhibitor cocktail (Roche, Switzerland). After 15 minutes of centrifugation at 12,000 rcf and 4°C, the supernatant from each sample was collected and boiled for 8 minutes. Each sample’s protein was electrophoretically separated on SDS-PAGE gels, transferred to PVDF membranes, and detected using antibodies against RGSV P3 and PC5.

### CUT&Tag assay

Nuclei of WT and RGSV-infested rice material were extracted using a cell nucleus isolation kit (Sigma, CELLYTPN1). The enrichment of genomic H3k9me3 modifications was carried out using the CUT&Tag kit (Vazyme, TD-903). The detailed steps are as follows: use a 100 μL of washing buffer to suspend about 1 × 10^5^ nuclei, incubate them with concanavalin A-coated magnetic beads, and the nuclei are tied to the magnetic beads. Add 1 μg of anti-H3K9me3 antibody (abcam, ab6002) or control antibody anti-H3 (abcam, ab1791) to 50 μL of antibody buffer and rotate slowly overnight at 4°C. Subsequently, the secondary antibody (diluted with wash buffer) was treated with 50 μL for an additional 1 h at room temperature. Treated nuclei were washed three times with DIG wash buffer to remove free antibodies and then further incubated with the highly active pA-Th5 transposase adapter complex (TD-903, Vazyme) to digest the host genome and obtain fragmented DNA. The DNA was purified by phenol-chloroform-isoamyl alcohol extraction and ethanol precipitation. 15 μL of DNA were mixed with 5μL of universal N5XX and uniquely coded N701 (TD-202, Vazyme), and then 12 cycles were performed in a PCR instrument to purify the amplification products. The size distribution of the libraries was examined by performing an Agilent 2100 analysis. Sequencing was performed using the Illumina HiSeq X-Ten platform (Novogene, Beijing, China). Sequencing of raw reads by filtering out sequencing adapters, short fragment reads and other low-quality reads. The clean reads were then mapped to the rice reference genome using Bowtie (version 0.12.8). Peak detection was performed using MACS2 (version 2.2.7.1; https://pypi.python.org/pypi/MACS/2.2.7.1).

### Statistical analysis

GraphPad Prism 9.0 (GraphPad Software Inc., USA: http://www.graphpad.com/) was used for the statistical analysis. All experimental data were examined using a Student’s t-test, a Mann Whitney test, or a Duncan’s multiple-range test.

## Results

### 1 RGSV infection triggers low levels of cytosine DNA methylation

**Figure 1:**
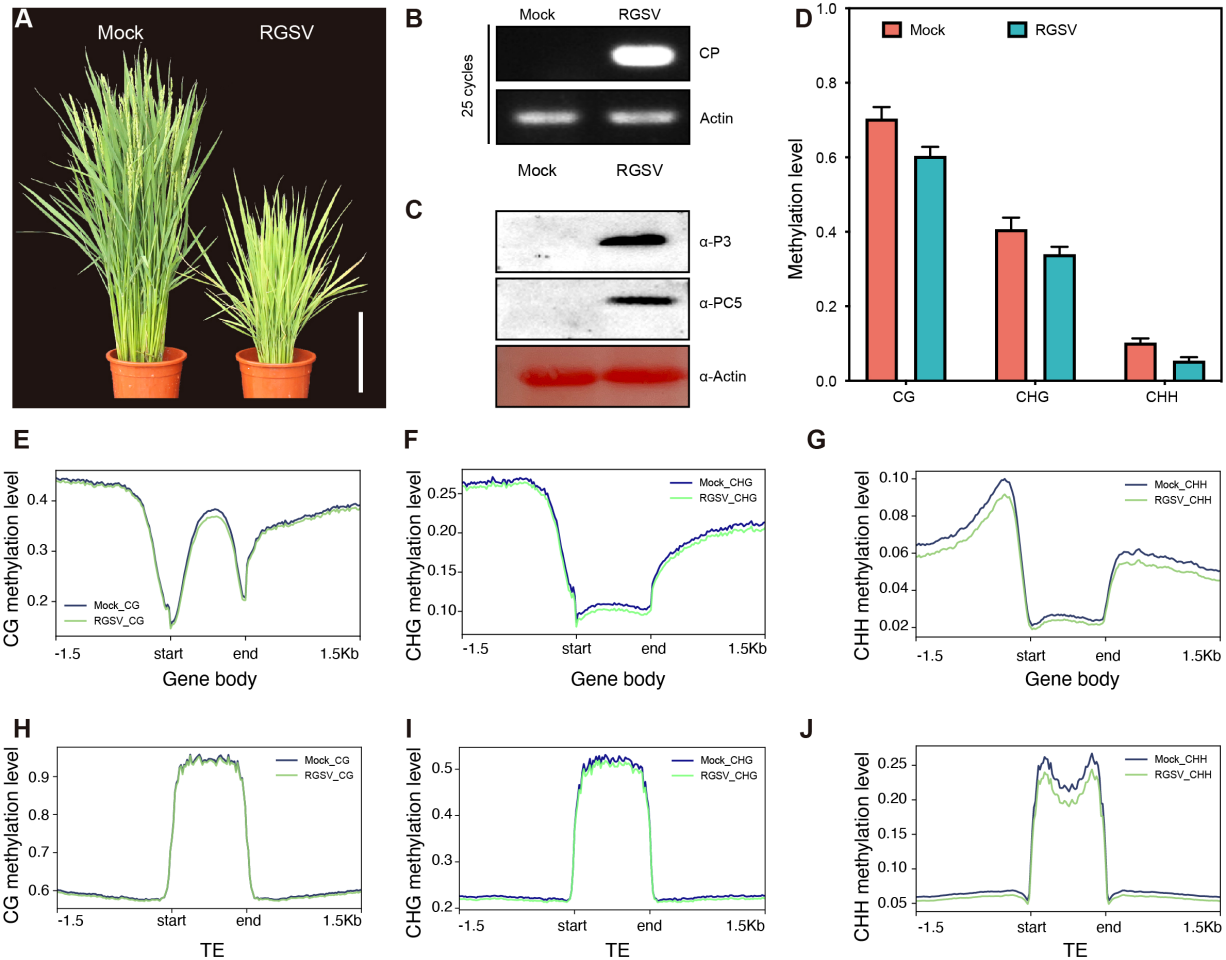
Characteristics of methylation genomics of rice infected with RGSV. **(A)** The signs of the disease induced by RGSV on rice plants. **(B)** The expression of the RGSV coat protein (CP) gene was measured using qRT-PCR. **(C)** The viral content in rice leaves infected with RGSV was detected using western blotting with anti-RGSV CP and anti-RGSV PC5 polyclonal antibody. Rice actin was used as the loading control. **(D)** The frequency histogram of methylation of CG, CHG and CHH types in RGSV infected and uninfected rice. **(E–J)** Visualization of DNA methylation profiles of mCG, mCHG and mCHH peripheral genes and transposable elements (TE_S_, within 1.5 kb) in RGSV-infected and uninfected rice.

Rice infected with RGSV exhibited grass-like dwarfism, increased tillers, narrow leaves, chlorotic yellowing, and many irregular brown rust spots. Most of the susceptible rice did not blossom and heading, occasionally produced short spikelets but not full grains (**Figure 1A**). Next, we performed qRT-PCR and western blot analysis to determine the accumulation of viral RNA in infected rice leaves. QRT-PCR revealed that the viral coat protein gene *PC5* was widespread in infected rice leaves (**Figure 1B**). Western blot analysis also revealed a high content of RGSV virulence protein P3 and coat protein PC5 (**Figure 1C**), indicating a high viral load in infected rice.

To evaluate and illustrate the impact of RGSV infection on the whole genome DNA cytosine methylation pattern of rice, one-month-old rice shoots infected with and without the virus were subjected to whole genome sulfite sequencing (BS). Overall, the average methylation levels of CG, CHG and CHH were about 62.8, 37.6 and 6.1% in rice leaves, and the levels of the three types of methylation in RGSV-infested rice leaves were slightly lower than those in normal rice (**Figure 1D**). When examining the distribution of CG, CHG, and CHH methylation in gene and TE regions, metaplots in genes revealed that the three types of methylation occurred more frequently in the flanking regions than in the gene body regions (**Figures 1E–G**). Near the transcription start and endpoints, DNA methylation is very low in the CG background, but progressively rises while leaving these sites. In contrast to gene body regions, TEs is highly methylated in all CG, CHG, and CHH sequences (**Figures 1H–J**) (Zemach et al., 2010), and it has been reported in the literature that DNA methylation in transposon silencing may be conserved in plants and is similar in Arabidopsis and soybean (Hsieh et al., 2009; Song et al., 2013). Following RGSV infestation, the genomic methylation levels of CG and CHG were almost indistinguishable, whereas CHH displayed hypomethylation, either in the gene body or in the TE region (**Figures 1G, J**), demonstrating that the effect of RGSV on DNA methylation in CG, CHG and CHH sequences was heterogeneous.

### 2 Dynamic DNA methylation alterations are focused in the promoter and intergenic regions

**Figure 2:**
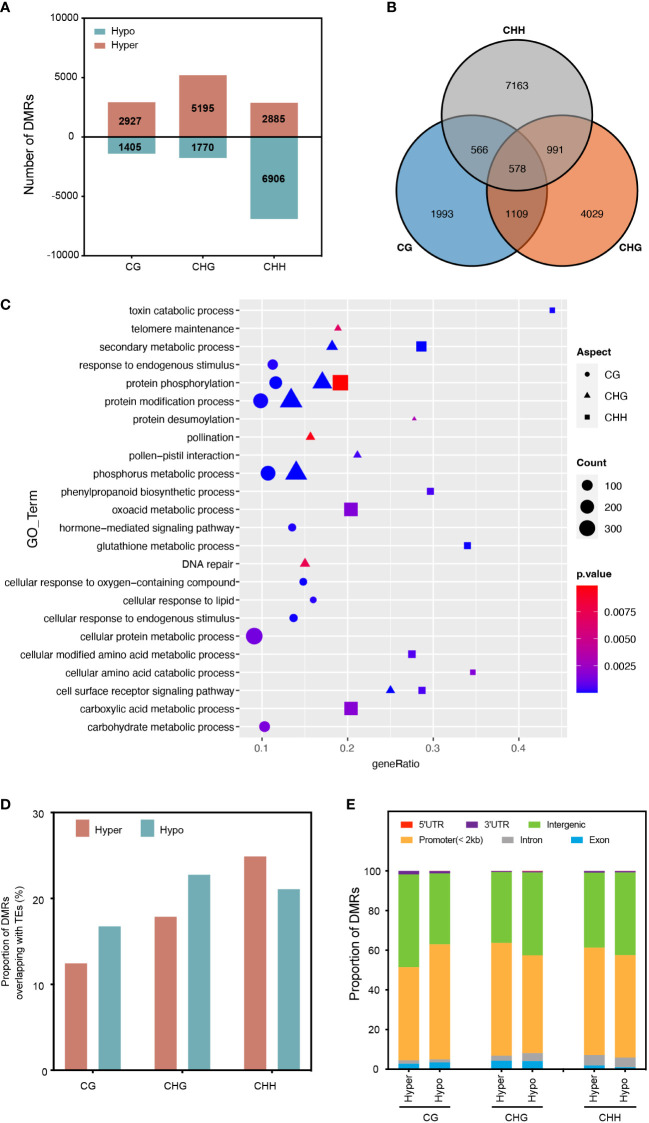
Characterization of differentially methylated regions (DMRs) in different cytosine contexts. **(A)** The number of DMRs near protein-coding genes in rice leaves after RGSV infection (within 1.5 kb). **(B)** Overlap of DMGs with different methylation contexts. **(C)** Gene Ontology (GO) analysis of DMGs from different methylation contexts. **(D)** The proportion of DMRs overlapping with transcriptional elements (TEs). **(E)** Genomic composition of DMRs surrounding genes.

To investigate the impact of RGSV infection on genomic DNA methylation in rice, we identified differentially methylated regions (DMRs) between mock and RGSV infections (DMR_S_; > twofold change, *P* < 0.05). Overall, 21088 DMRs were identified for the 2 kb region close to the expressed gene by comparing the RGSV-infected and uninfected databases (**Table S2**). Most of the DMRs occurred in the CHH context, and the number of CHH hypo-DMRs was approximately twice that of hyper-DMRs. However, DMRs in hyper-CG were more than 2-fold higher than those in hypo-CG, and what’s more, DMRs in hyper-CHG are almost three times as large as those in hypo-CHG (**Figure 2A**). Only 578 DMR-related genes (DMGs) are linked with all three forms of DNA methylation, while 62.5 percent of DMGs in DNA methylation alteration triggered by RGSV infestation are associated with only one cytosine context (**Figure 2B**). A gene ontology (GO) category analysis was undertaken to better understand the biological functions of DMGs in diverse contexts. Whether highly methylated or demethylated, 578 DMGs linked with three forms of DNA methylation were discovered to be mostly engaged in protein phosphorylation, phosphorus metabolism, protein polyubiquitination, and toxin catabolic pathways (**Figure S1**). CHH-DMGs are individually involved in the biosynthesis of phenylpropane, glutathione metabolism, and cell-modified amino acid metabolism, and CHH-DMGs and CHG-DMGs are jointly associated with secondary metabolic processes and the cell surface receptor signaling pathway (**Figure 2C**). Next, we evaluated the distribution of CG-DMRs, CHG-DMRs and CHH-DMRs in the genome and found that both hyper- and hypo-DMRs overlapped moderately with TE bodies (about 20%) (**Figure 2D**). For the different regions of the genes, the vast majority are distributed in the promoter and intergenic regions. Moreover, the distribution of CHH-DMRs in exons was extremely small, with only 1.89% (hyper-DMRs) and 0.98% (hypo-DMRs) (**Figure 2E**). In a nutshell, the majority of the RGSV-induced DMR near genes were positioned in the context of CHH, with the vast majority located in the promoter and intergenic regions, and moderately overlapping with TEs.

### 3 Analysis of RGSV pathogenicity-associated genes by integrating DNA methylation and transcriptome

**Figure 3:**
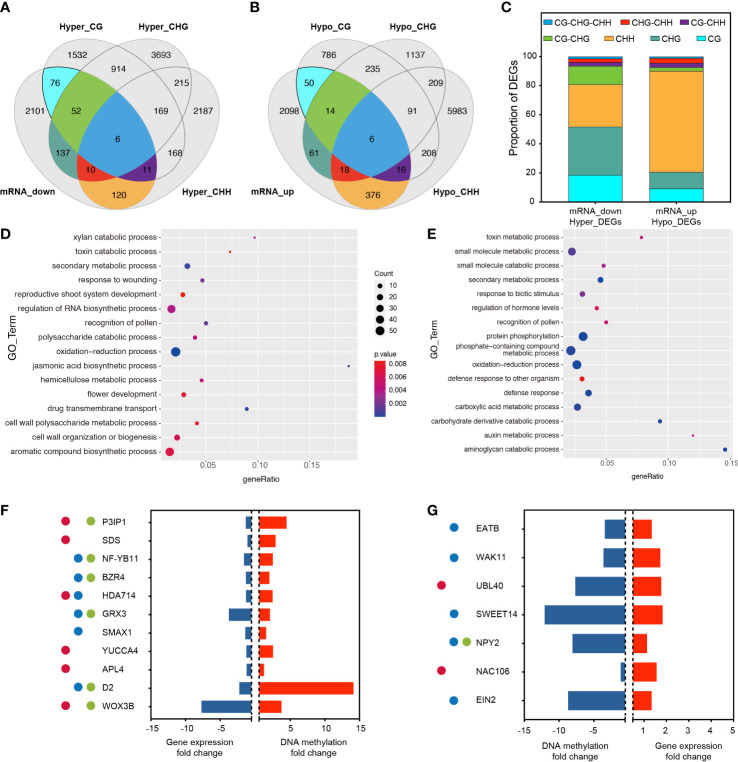
Association analysis of genes with changes in DNA methylation and transcript abundance. **(A)** Venn diagram showing the association between transcriptionally down-regulated DEGs and the three hypermethylated types of DMGs. **(B)** Venn diagram showing the association between transcriptionally up-regulated DEGs and the three hypomethylated types of DMGs. **(C)** Proportion of overlapping DMGs in different methylation contexts. **(D)** GO analysis of overlapping DMGs in mRNA_down and Hyper_DEG_S_. **(E)** GO analysis of overlapping DMGs in mRNA_up and Hypo_DEG_S_. **(F)** After RGSV infection of rice, most of these genes were associated with fertility (●), plant height (●), and tillering (●), and these genes were down-regulated in the transcriptome (blue bars) while remaining hypermethylated (red bars). P3IP1 (LOC_Os02g33680); SDS (LOC_Os03g12414); NF-YB11 (LOC_Os08g07740); BZR4 (LOC_Os02g13900); HDA714 (LOC_Os12g08220); GRX3 (LOC_Os01g13950); SMAX1 (LOC_Os08g15230); YUCCA4 (LOC_Os01g12490); APL4 (LOC_Os07g13980); D2 (LOC_Os01g10040); WOX3B (LOC_Os05g02730). **(G)** After RGSV infection of rice, most of these genes were associated with fertility (●), plant height (●), and tillering (●), and these genes were up-regulated in the transcriptome (red bars) while remaining hypomethylated (blue bars). EATB (LOC_Os09g28440); WAK11 (LOC_Os02g02120); UBL40 (LOC_Os09g31031); SWEET14 (LOC_Os11g31190); NPY2 (LOC_Os06g08550); NAC106 (LOC_Os08g33670); EIN2 (LOC_Os07g06130).

To determine whether promoter-related DMR_S_ induced by RGSV infestation affects gene expression, RNA-Seq was performed using the same material (rice leaves) as for methylation analysis. Analysis of the correlated transcriptome and methylation data showed that 2513 gene expression has been lowered, and 2639 gene expression has been raised (**Figures 3A, B**, **Table S3**). Among the 2,513 down-regulated genes, 412 genes were hypermethylated, and among the genes transcriptionally upregulated, 541 genes showed hypomethylation. Among the down-regulated and hypermethylated genes, single CG, CHG and CHH type methylation accounted for a relatively large proportion, 18.45%, 33.25% and 29.13%, respectively (**Figure 3C**). These genes are mainly involved in aromatic compound biosynthetic process, cell wall organization or biogenesis, flower development and regulation of RNA biosynthetic process (**Figure 3D**). Among the upregulated and hypermethylated genes, a single CHH type methylation accounted for the largest proportion of genes, reaching 69.50% (**Figure 3C**). This type of gene is mainly involved in phosphate-containing compound metabolic processes, protein phosphorylation, carboxylic acid metabolic processes and small molecule metabolic processes (**Figure 3E**).

Among the down-regulated and hypermethylated genes, we analyzed and identified 11 transcripts involved in fertility, plant height and tillering, including P3IP1 (RGSV P3-Inducible Protein 1), SDS (Solo Dancers), NF-YB11 (Nuclear Transcription Factor Y Subunit B 11), BZR4 (Brassinazole Resistant 4), HDA714 (Histone Deacetylase 10), GRX3 (cc-Type Glutaredoxin 3), SMAX1 (Suppressor of Max 2 1), YUCC4, APL4 (ADP-Glucose Pyrophosphorylase), D2 (Dwarf2), WOX3B (Wuschel-Like Homeobox 3B). These transcripts were significantly hypermethylated at the CpG site of the same gene, while gene expression showed significant down-regulation, and qRT-PCR data also confirmed that the gene expression almost exactly matched with the transcriptome data (**Figure S2**). Six transcripts were involved in pollen abortion, reduced fruit set and reduced fertility, six mutants were involved in plant dwarfing process and six transcripts were involved in regulation of tiller number and tiller angle (**Figure 3F**). Among the transcriptionally up-regulated and hypermethylated genes, we analyzed and identified seven transcripts involved in fertility, plant height and tillering, including EATB (Ethylene-Response AP2/ERF Factor), WAK11 (Wall-Associated Receptor Kinase 11), UBL40 (Ubiquitin Fusion Ribosomal Protein L40), SWEET14, NPY2 (Small GTP-binding Protein), NAC106, EIN2 (Ethylene Insensitive 2). Among them, two transcripts were involved in fertility, five transcripts were involved in plant height regulation, and one transcript was involved in the regulation of tiller number and tiller angle. These transcripts underwent significant hypomethylation at the CpG site of the same gene, while gene expression showed significant up-regulation (**Figure 3G**).

### 4 Altered H3K9me3 modification triggered by RGSV infestation activates the expression of BR- and CK-related genes

**Figure 4:**
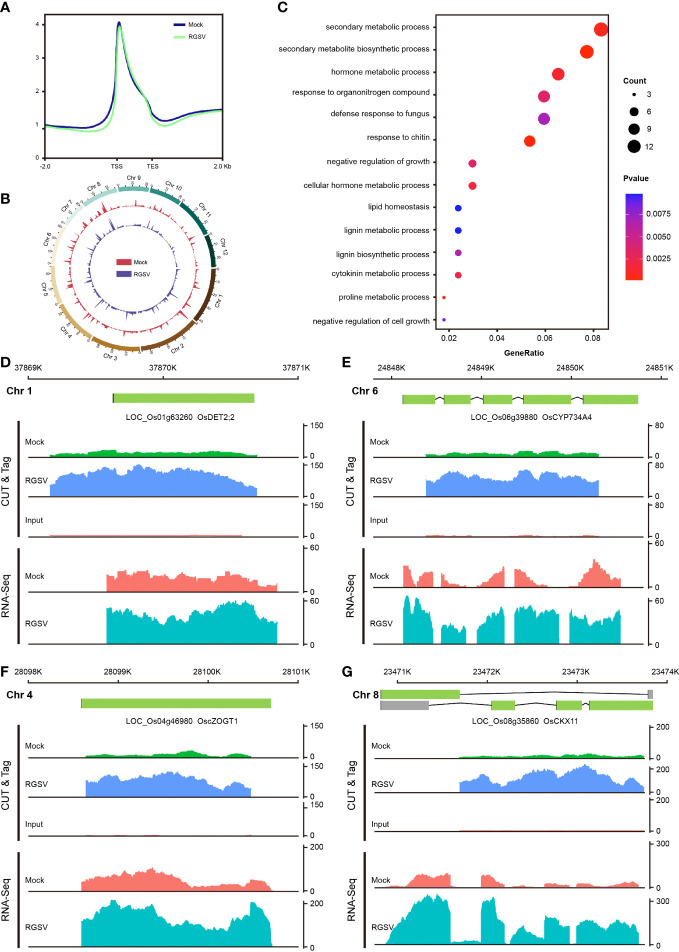
Genome-wide mapping of H3K9me3 and expression of its related genes after RGSV infection in rice. **(A)** Signal intensity of H3K9me3 in the TTS and TES intervals and its upstream and downstream 2kb. **(B)** Distribution of H3K9me3 in the whole rice genome. **(C)** GO enrichment analysis results of differential genes. **(D–G)** Gene browser view of multiple genes in CUT&Tag and RNA-seq.

H3K9me3, one of the hallmarks of heterochromatin, has been extensively and intensively studied in mammalian cells, with most of its studies being a repressive histone modification, when a few works have also found H3K9me3 to be associated with gene transcriptional activation. Our previous study found that H3K9me3 is also widely distributed in plants, but little is known about its function. CUT&Tag assay and high-throughput sequencing were carried out on WT and RGSV-infected rice samples to detect the modification difference of histone H3K9me3. The signal intensities of the H3K9me3-labeled 2 kb upstream and downstream of the genome-wide transcriptional initiation site (TSS) and transcriptional termination site (TES) of the two samples are shown in **Figure 4A**, and there is a main peak near the TSS. The genome-wide distribution of H3K9me3 modifications in WT and RGSV-infected rice samples is shown in **Figure 4B**, indicating that H3K9me3 modification is widely distributes in the rice genome. Peaks containing the H3K9me3 mark were found in Mock and RGSV-infected samples using MACS2 with the numbers of 8081 and 10729, respectively. The width range of the peaks of both samples was counted, and some regions were found to be wider than 10,000 bp (**Figure S3A**). The median width of the H3K9me3-labeled region in both samples was around 1000 bp (**Figure S3B**), indicating that the H3K9me3 mark is a broad modification. The genomic regions where the peaks of both samples were located were annotated, and it was found that about 90% of the peaks were located in the gene promoter (≤1kb) region (**Figures S3C, D**), and since the H3K9me3 mark width was generally >1000 bp, H3K9me3 was also mainly located in the gene body region. These results imply that H3K9me3 mark may be closely related to gene expression. The software MAnorm was used to find the different H3K9me3 modifications in the two samples, and a total of 500 peaks with significant differences were found (**Figure S3E**). There were 391 peaks with significantly increased H3K9me3 modification and 109 peaks with significantly decreased in the RGSV-infected samples compared to the WT samples. There were 499 genes associated with these 500 peaks (Figure SF), and a scatter plot of the number of differences between all peaks and their associated genes in the two samples was made and the correlation coefficient was calculated to be 0.27 (**Figure S3G**), indicating a positive correlation between H3K9me3 modifications and gene expression.

The 499 genes associated with 500 peaks were subjected to GO and KEGG pathway analyses, and the GO analysis showed that these genes were mainly involved in hormone metabolic process (GO:0042445), cytokinin metabolic process (GO:0009690), negative regulation of growth (GO:0045926), and so on (**Figure 4C**). KEGG pathway analysis showed that these genes were mainly concentrated in the metabolic pathways of phenol propane biosynthesis, zeatin biosynthesis (dosa00908), vitamin B6 metabolism (dosa00750), and brassinosteroid biosynthesis (dosa00905) and other metabolic pathways (**Figure S3H**). Four genes related to zeatin and brassinosteroid biosynthesis were selected among the entries of the KEGG pathway, including LOC_Os04g46980 (cZOGT1), LOC_Os08g35860 (CKX11), LOC_Os01g63260 (DET2;2) and LOC_Os06g39880 (CYP734A4). Analysis of the gene browser view of these gene expression levels in relation to the enrichment of H3K9me3 modifications revealed that H3K9me3 modifications were mainly concentrated in the gene body region, and when the modification enrichment increased, its corresponding gene expression also increased. In short, H3K9me3-labeled genes tend to show high expression levels, but the molecular mechanisms underlying the specific role of H3K9me3 modifications need to be explored in depth.

## Discussion

An integrated multi-omics approach has been used to identify and decipher stress resistance and disease immune responses in economically important crops, including wheat, soybean and rice, and to build on this to investigate genes that control important agronomic traits (Deshmukh et al., 2014; Talukdar and Sinjushin, 2015; Shah et al., 2018; Wang et al., 2019a; Tang et al., 2021). In the field of viruses, integrated multi-omics analysis to reveal the mechanisms of viral perturbation to the host has been extensively and intensively studied in the animal and medical fields. In particular, since the COVID-19 outbreak, papers on the integrated multi-omics analysis of its infection mechanism have mushroomed, and researchers have screened a variety of enzyme inhibitor drugs with significant antiviral effects (Su et al., 2020; Lam et al., 2021; Stukalov et al., 2021). In contrast, the multi-omics integration analysis for plant viruses is still in its infancy, and only a few relevant studies have been reported. A study has been conducted to identify host proteins and key genes interacting with Turnip mosaic virus (TuMV) P1 by proteomics and transcriptomics, dissecting the regulatory network of P1/HC-Pro-mediated post-transcriptional gene silencing (PTGS) repression of TuMV from a four-dimensional perspective (gene expression, gene correlation, location and time course) (Hu et al., 2020). Recent studies have also shown multiplex analysis of the interaction between potato virus Y (PVY) and potato tolerance. To fully capture the complexity of interactions, studies capture changes at the molecular level at various levels, including the transcriptome, small RNAome, degradome, proteome, and hormoneome, supplemented by the measurement of virus accumulation, photosynthetic activity and symptom characterization, this provides a multi-level view for fine-tuning the response of potato to virus infection (Stare et al., 2019).

Rice is one of the world’s most important food crops, and its production is critical to food security. Rice virus diseases transmitted by vector insects are a serious threat to rice production. A recent work, through six years of continuous research in many locations, found that a variety of rice viruses can cause different degrees of damage to almost all important rice germplasm in China. The germplasm tested included japonica, indica, and hybrid rice as well as sterile and restorative lines, which generally lacked broad-spectrum resistance to rice viruses (Wu et al., 2022). These results indicate that none of the currently cultivated rice germplasm has broad-spectrum resistance to rice viruses, and once these rice viruses break out, it will seriously reduce rice yield and affect food security. There are 19 reported rice virus diseases in the world, 10 of which are serious threats to rice production (Sasaya et al., 2014). Among them, RGSV is transmitted by the vector insect brown planthopper in a persistently proliferative manner (Hibino et al., 1985), and was first discovered in the Philippines in 1963 (Rivera et al., 1966). At present, RGSV is still sporadically distributed in Guangdong, Guangxi, Hainan and other provinces in China, and there is a potential for outbreaks to endanger food security at any time. Previous work has identified that RGSV-encoded P3 activates the rice E3 ubiquitin ligase P3IP1, which targets and degrades Pol IV, linking the viral pathogenesis process to the ubiquitin-proteasome system (UPS), but our knowledge of the overall perception and dynamic association of RGSV infestation and pathogenesis is still superficial. In this project, we applied multi-omics, including epigenome, transcriptome and histone modification, to more comprehensively reflect the dynamic changes of rice triggered by RGSV infection at a multi-dimensional level. Preliminary excavate the key genes for dwarfism, multiple tillering and low fertility in rice caused by RGSV pathogenesis, so as to lay a solid foundation for subsequent in-depth studies.

H3K9me3 labeling has been widely studied in animal cells, which is mainly concentrated in heterochromatin and acts as a transposon silencing (Nicetto and Zaret, 2019). In this study, we found that the H3K9me3 modification is widely present in rice and is a broad type of modification, mainly concentrated in the gene promoter (≤1kb) and gene body region, which is closely related to gene expression (**Figures S2C, D**). In animal, H3K9me3 is mainly related to the formation of heterochromatin, which is widely distributed in retrotransposons and some gene promoters. H3K4 methylation is widely distributed in euchromatin and is associated with actively transcribed genes in plants and animals (Wang et al., 2009; Liu et al., 2010; Roudier et al., 2011; Shilatifard, 2012; Local et al., 2018). H3K4me1 was distributed at a much lower level in the gene body region than H3K4me2 and H3K4me3. H3K4me2 covered the entire gene body region with a weak peak near the transcription start site (TSS), whereas H3K4me3 was enriched near the TSS region with a sharp peak, and H3K36me3 is mainly distributed at the 5’ end of the gene body (Local et al., 2018). In terms of the enrichment of the modifications, the H3K9me3-tagged genes tended to show high expression levels (**Figures S2F, G**). In mammals, several works have confirmed the H3K9me3 association with the transcribed regions of some active genes (Vakoc et al., 2005; Squazzo et al., 2006; Vakoc et al., 2006). However, it remains to be determined whether H3K9me3 could serve different outcomes depending on genomic and/or chromatin context, and whether it plays a direct or indirect role in plant transcriptional regulation. After RGSV infection, H3K9me3 modification changed significantly at the whole genome level, affecting the expression of some genes related to hormone pathway, such as CTK (cZOGT1, CKX11) and BR (DET2;2, CYP734A4) (**Figures 4D–G**). Among them, cZOGT1 is one of the genes encoding cis-Zeatin-O-glucosyltransferases, and overexpression of cZOGT1 results in a dwarf phenotype in rice (Kudo et al., 2012). OsCKX11 encodes a cytokinin oxidase, which is responsible for the degradation of cytokinin. Knockdown of OsCKX11 can increase both photosynthesis and grain number to improve crop yield (Zhang et al., 2021). While OsDET2;2 and CYP734A4 are key enzymes of BR synthesis and degradation pathways, respectively (Qian et al., 2017), altered expression of these genes may lead to abnormal development in rice. Regarding the altered H3K9me3 modification, it may be directly related to changes in methyltransferases and demethylases, such as the P6 protein of Cauliflower mosaic virus (CaMV) that interacts with the histone deacetylase Histone Deacetylase 2C (HD2C) to increase genome-wide acetylation levels (Li et al., 2021). There are few studies on the methyltransfer and demethylases of H3K9me3 in plants, and the known H3K9me3 methyltransferase in rice is SET Domain Group 728 (SDG728) and the demethylase is JMJ706 (Sun and Zhou, 2008; Qin et al., 2010). We will follow up with viral proteins encoded by RGSV (e.g., virulence protein P3) to investigate whether there are interactions with histone methyltransferases and demethylases.

## Data availability statement

The data presented in the study are deposited in the NCBI database under following accession number: PRJNA891900.

## Author contributions

XW, HL, XJ, CC and TT performed the experiments and analyzed the data. SZ, SSZ and JW designed the research. BL, XD and JH provided technical support and conceptual advice. XW, SZ and JW wrote the manuscript. All authors contributed to the article and approved the submitted version.

## Supplementary material

Supplementary Figure 1 GO analysis of 528 overlapping DMRs from three types of DNA methylation contexts.

Supplementary Figure 2 qRT-PCR analysis of the expression of related genes after RGSV infestation in rice Student’s t test, **P* < 0.05, ***P* < 0.01 and ****P* < 0.001. Data are represented as means ± SD, n=5.

Supplementary Figure 3 Genome-wide characterization of H3K9me3 modification. **(A)** The width range of all H3K9me3 modification. **(B)** Distribution of H3K9me3 modification within 2000 bp. **(C, D)** H3K9me3 annotation for the region where the tag is located. **(E)** Differential H3K9me3 labeled region. **(F)** Differential expression of H3K9ME3related genes. **(G)** Correlation between H3K9me3 modification and related gene expression. **(H)** Results of KEGG pathway analysis of differentially expressed genes.

Supplementary Table 1 Oligonucleotides Used in This Study.

Supplementary Table 2 Differentially methylated regions (DMRs) in rice after RGSV infection.

Supplementary Table 3 Differentially expression genes (DEGs) in rice after RGSV infection.
