# Supplementary figures and images for: Genome-wide analysis of epigenetic and transcriptional changes in the pathogenesis of RGSV in rice
Source: Front Plant Sci. 2023 Jan 11;13:1090794. doi: 10.3389/fpls.2022.1090794 (PMC9874293; doi:10.3389/fpls.2022.1090794)

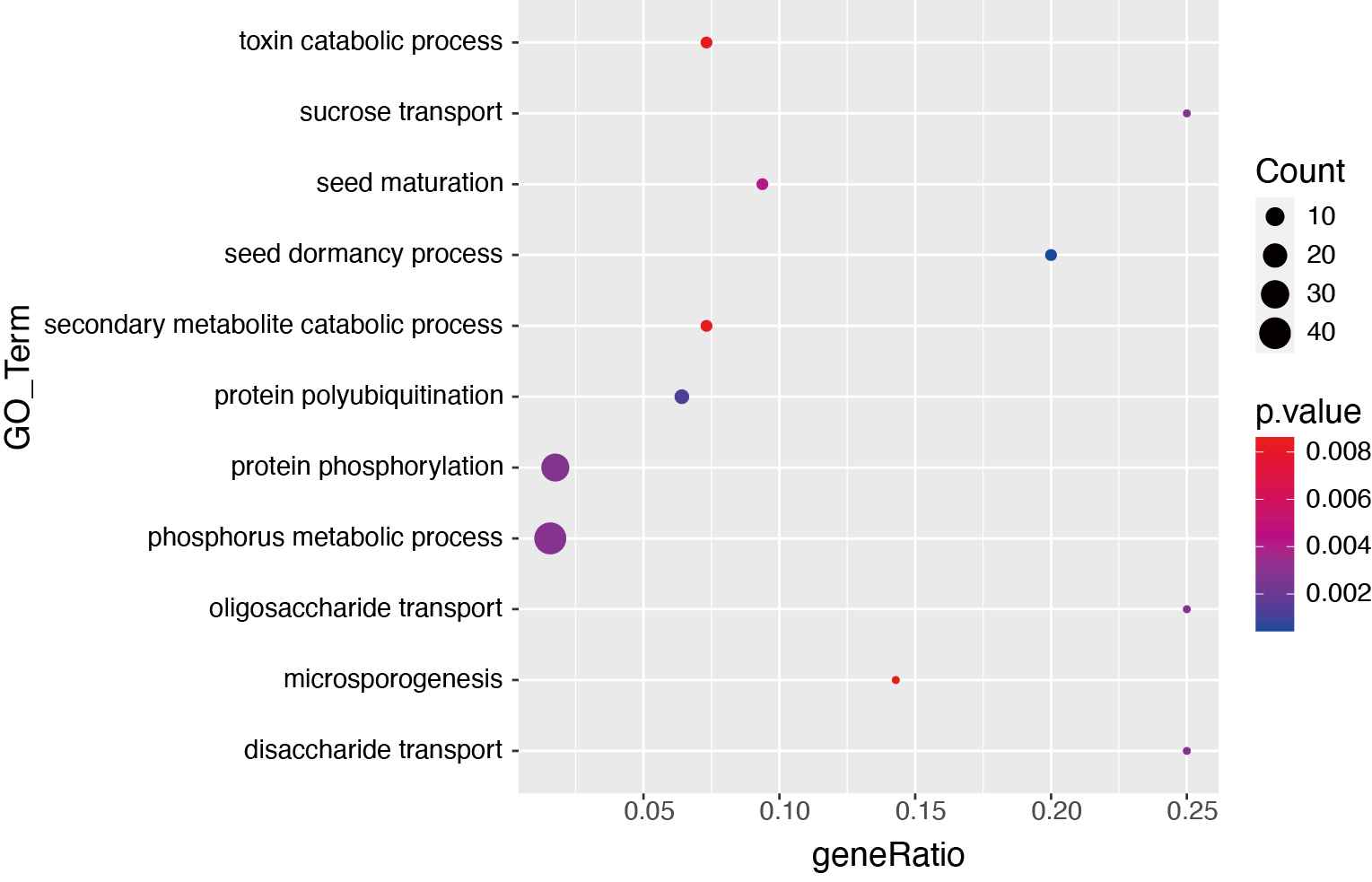

Supplement: Supplementary Figure 1 — GO analysis of 528 overlapping DMRs from three types of DNA methylation contexts. [file Image_1.jpeg]

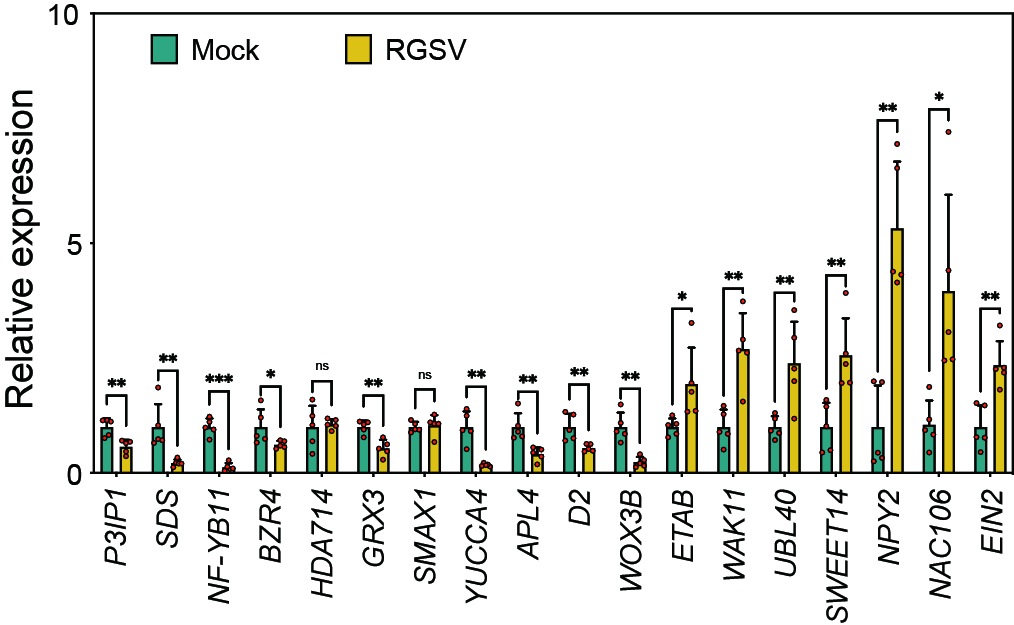

Supplement: Supplementary Figure 2 — qRT-PCR analysis of the expression of related genes after RGSV infestation in rice Student’s t test, *P < 0.05, **P < 0.01 and ***P < 0.001. Data are represented as means ± SD, n=5. [file Image_2.jpeg]

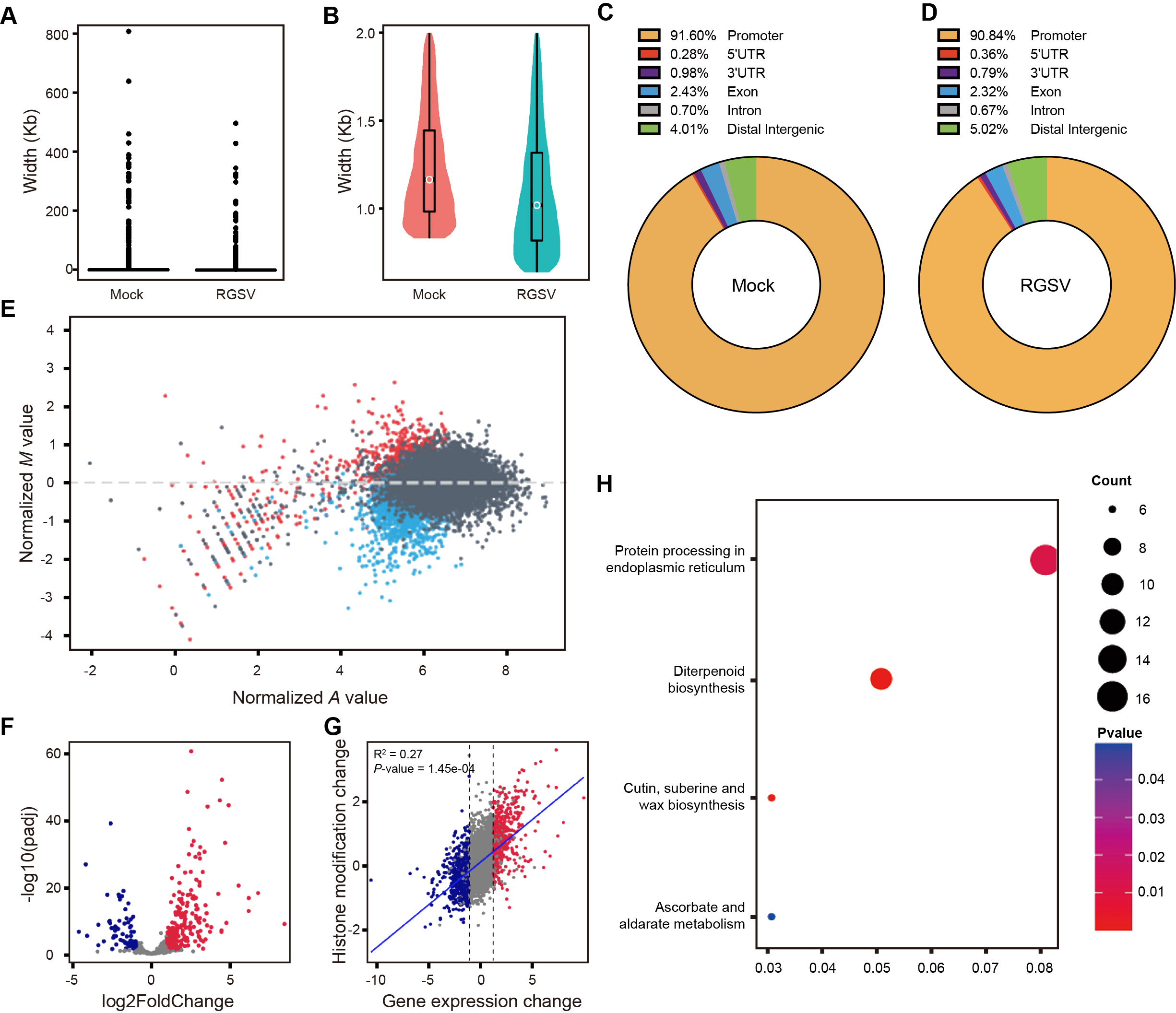

Supplement: Supplementary Figure 3 — Genome-wide characterization of H3K9me3 modification. (A) The width range of all H3K9me3 modification. (B) Distribution of H3K9me3 modification within 2000 bp. (C, D) H3K9me3 annotation for the region where the tag is located. (E) Differential H3K9me3 labeled region. (F) Differential expression of H3K9ME3related genes. (G) Correlation between H3K9me3 modification and related gene expression. (H) Results of KEGG pathway analysis of differentially expressed genes. [file Image_3.jpeg]
